# Incorporating patient-reported outcome measures into routine paediatric pharmacovigilance: opportunities for safety monitoring with practical use scenarios from paediatric oncology

**DOI:** 10.3389/fphar.2025.1719963

**Published:** 2026-02-09

**Authors:** Pedro Teodoro, Ricardo M. Fernandes, Inês Ribeiro, Renato Ferreira-da-Silva

**Affiliations:** 1 Faculty of Pharmacy of the University of Coimbra, Coimbra, Portugal; 2 Clinical Pharmacology and Therapeutics Laboratory, Faculdade de Medicina da Universidade de Lisboa, Lisboa, Portugal; 3 Porto Pharmacovigilance Centre, Faculty of Medicine of the University of Porto, Porto, Portugal; 4 RISE-Health, Department of Community Medicine, Information and Health Decision Sciences, Faculty of Medicine of the University of Porto, Porto, Portugal

**Keywords:** drug-related side effects and adverse reactions, paediatrics, patient reported outcome measures, patient safety, patient-centred care, pharmacovigilance

## Abstract

**Background:**

Traditional pharmacovigilance systems often fail to capture children’s experiences of adverse events (AEs), particularly subjective symptoms that affect daily functioning and quality of life. Patient-Reported Outcome Measures (PROMs) offer a complementary perspective by enabling direct input from children or their caregivers on treatment-related outcomes.

**Objectives:**

To examine how PROMs can be integrated into routine paediatric pharmacovigilance and to propose practical use scenarios that illustrate their potential applications.

**Methods:**

We conducted a targeted narrative review using MEDLINE (via PubMed), Scopus, and Web of Science, complemented by manual searches of regulatory guidance and reference lists. Studies were included if they addressed the use of PROMs in paediatric contexts relevant to pharmacovigilance. Extracted data were synthesised across domains, including measurement properties, regulatory uptake, and potential for safety monitoring. The search was carried out in June 2025.

**Results:**

PROMs have demonstrated strong validity, feasibility, and relevance for detecting symptomatic AEs and monitoring health-related quality of life in paediatric populations. Instruments such as PROMIS Paediatric and other condition-specific tools have been successfully used across clinical, regulatory, and research settings. PROMs enhance the detection and characterisation of AEs by capturing dimensions not readily assessed through traditional spontaneous reporting. Building on these findings, we present five practical use scenarios that exemplify how the Ped-PRO-CTCAE can be adapted for real-world safety monitoring in pharmacovigilance workflows, including spontaneous reporting, EHR-based monitoring, pharmacy-based follow-up, and medication rechallenge.

**Conclusion:**

PROMs offer a structured, patient-centred approach to paediatric pharmacovigilance, enhancing post-marketing safety monitoring through systematic symptom reporting.

## Highlights

- PROMs can help address underreporting of symptomatic adverse events in routine paediatric pharmacovigilance by providing structured, patient-centred information.
- PROMs may be integrated into various routine pharmacovigilance workflows, including spontaneous reporting, EHR-based monitoring, pharmacy-based follow-up, and medication rechallenge. We illustrate these opportunities through five original paediatric oncology use scenarios.
- Successful implementation requires sufficient contextual data in initial reports to support the selection of appropriate PROM item selections and the interpretation of symptoms.
- Routine integration of PROMs still faces operational barriers such as technological limitations, the need for staff training, and the lack of seamless data transmission to pharmacovigilance centres.

## 1 Introduction

In recent decades, advances in medical treatments and a decline in infectious diseases have significantly increased survival rates among children. However, this progress has led to a growing number of children living with chronic illnesses, requiring extended therapeutic management. This increasing prevalence of chronic conditions often leads to multimorbidity and subsequent polymedication, further complicating treatment regimens and potentially increasing the risk of adverse events (AEs) (Kish et al., 2018; Perrin and Gortmaker, 2007).

Children are at heightened risk of experiencing AEs, partly because many medications prescribed to this population have been approved with limited evidence regarding their safety and efficacy in children (Kongkaew et al., 2008). Paediatric Randomized Clinical Trials (RCTs) are relatively scarce, and available data often provide insufficient information about the long-term impacts of medications on children. Systematic reviews have shown that the average incidence of AEs in paediatric patients hovers around 9.5% (Impicciatore et al., 2001; Khan et al., 2020). AEs have been reported to account for nearly 5% of all hospital admissions in paediatric patients (Angamo et al., 2016) and represent a substantial cause of hospitalisations among children, comprising 7.3% of total admissions at the hospital (Mathur et al., 2022). Moreover, a review of six prospective studies of AEs in childhood estimated that the incidence of AEs was 10.9% (95% CI 4.8, 17.0) in hospitalized children (Clavenna and Bonati, 2009), suggesting that the frequency of AE reported in hospitalized children has remained consistent since 2001.

AEs in paediatric patients have been demonstrated to result not only in hospital admissions or prolonged hospital stays but also in long-term disabilities or, in some cases, death (Le et al., 2006). Unfortunately, there is limited data on mortality rates linked to AEs in paediatric populations, which highlights the need for more comprehensive studies on this critical aspect. The majority of AEs (63.89%) were classified as unpredictable and not dose-related, with the most events considered probable (87.5%), moderate in severity (51.39%), and preventable (88.89%) (Mathur et al., 2022). To further characterize the scope of the issue, AEs and AE-related hospital admissions can be analyzed based on factors such as patient age, drug classes involved, affected organ systems, gender distribution, and causality assessment (Mathur et al., 2022; Neininger et al., 2022; Balidemaj, 2021). These findings underscore the complexity and significance of AEs in paediatric populations, highlighting the need for continued research and vigilance in this area (Mathur et al., 2022; Neininger et al., 2022; Balidemaj, 2021; Wu et al., 2019; Mueller et al., 2020).

Consequently, the need for pharmacovigilance of paediatric medicines has become increasingly critical, particularly as new or repurposed drugs are introduced into paediatric care (Bouquet et al., 2018). The inherent limitations of RCTs in generating relevant safety signals and the challenges of extrapolating safety data from the adult population to paediatric patients highlight the need for continuous monitoring and targeted research in this vulnerable demographic (Neubert, 2012). Strengthened pharmacovigilance efforts can facilitate the identification and mitigation of AEs in children, ultimately contributing to safer and more effective therapeutic interventions (Narmada et al., 2021; Bodda Deepthi Rani et al., 2024). Spontaneous reporting remains the primary method for identifying potential AEs safety signals (Waller, 2006; López-Valverde et al., 2021). However, it often captures only a small subset of all AEs that occur, particularly those that significantly impact the child’s quality of life. Many other AEs, though present, may go unnoticed or are not recognised as drug-related, potentially leading to significant underreporting. This underscores the growing need for active pharmacovigilance strategies, which involve more robust and systematic methods in post-marketing surveillance (Nasso et al., 2020; Dittrich et al., 2022; Lombardi et al., 2018; Menniti-Ippolito et al., 2000).

To ensure effective post-marketing surveillance, particularly in paediatric populations, validated tools are essential for capturing patient experiences. Patient-Reported Outcomes (PROs), specifically Patient-Reported Outcome Measures (PROMs), have emerged as critical instruments in this context. PROMs are structured tools designed to gather data directly from patients or their caregivers. They provide valuable insights into the child’s real-world experience with their health, including perceptions of their health status, quality of life, and the outcomes of any treatments received. By capturing subtle and significant AEs, PROMs provide a patient-centred approach to pharmacovigilance (Almeida et al., 2024). Among these instruments, the Patient-Reported Outcomes Measurement Information System (PROMIS) Paediatric measures stand out, having been globally validated for their ability to characterise disease and treatment experiences from the perspective of affected youth (Weitzman et al., 2023). However, the incorporation of PROMs into routine clinical pharmacovigilance practice remains limited globally, with their use often restricted to clinical practice or pharmacoepidemiological studies rather than systematic safety monitoring in real-world practice. This highlights the need to review their current applications in pharmacovigilance and explore how they can be more effectively integrated into routine pharmacovigilance activities.

This article critically explores the use of PROMs in paediatrics for pharmacovigilance, focusing on their role in monitoring medicine safety and discussing the associated challenges and implications. It also presents four practical use scenarios, each accompanied by a clinical case, to demonstrate how a selected PROM can be applied to routine safety monitoring and integrated into pharmacovigilance workflows. By examining the potential for PROMs to provide more comprehensive and higher-quality data on AEs, this article highlights their role in enhancing pharmacovigilance systems and improving regulatory and clinical decision-making for paediatric medicines.

## 2 Methods

### 2.1 Search strategy and data sources

The research team conducted a comprehensive literature search to identify articles containing information on the use of PROMs in paediatric populations, regardless of clinical context or original purpose, with potential applicability to pharmacovigilance. We searched MEDLINE (via PubMed), Scopus, and Web of Science in June 2025. Both MeSH terms and free-text keywords were used, including patient-reported outcomes, patient-reported outcome measures, PROMs, adverse drug reactions, adverse events, drug safety, post-marketing surveillance, pharmacovigilance, paediatrics, children, and medicines. Boolean operators (AND/OR) were applied to refine the search. We manually searched the reference lists of selected articles using a snowballing strategy to identify additional relevant studies, while also considering unpublished sources, such as preprints and publicly available technical reports from regulatory agencies.

The search strategy followed the main steps for narrative reviews outlined by Baethge et al. in the SANRA scale, a tool for the quality assessment of narrative review articles (Baethge et al., 2019). This tool evaluates key domains, including the justification of the review’s relevance, clarity of objectives, adequacy of the literature search, appropriateness of referencing, and balanced scientific reasoning, while recognising the inherent limitations in reproducibility.

### 2.2 Study selection and eligibility criteria

The study selection was conducted between June and August 2025, based on its relevance to PROMs in paediatric settings, where the tools could potentially be used to monitor or characterise AEs or safety concerns. Eligible studies included methodological articles, clinical studies, and other research evaluating the development, validation, or application of PROMs in paediatric populations. No exclusion criteria were applied with respect to study design, publication language, year of publication, funding source, or geographical setting.

### 2.3 Screening and data extraction

Two reviewers independently screened titles and abstracts for eligibility, followed by a full-text assessment of potentially relevant articles. Discrepancies were resolved through discussion and consensus. Data extraction was performed thematically, capturing information on the purpose and scope of each PROM, target age groups, domains assessed, validation methods, and reported or potential applications in monitoring AEs and supporting pharmacovigilance processes in paediatric populations.

## 3 Review and perspectives

### 3.1 Concept and use of PROMs in regulatory and clinical decision-making

Originally developed to assess the impact of treatment in fields such as oncology, where quality of life is a primary concern, PROMs have since evolved into indispensable tools in various healthcare settings. They are now routinely used in RCTs and pharmacoeconomic studies to assess patient-centred outcomes. Regulatory bodies such as the Food and Drug Administration (FDA) and the European Medicines Agency (EMA) increasingly incorporate PROM data in their drug approval processes, recognising the importance of patient perspectives (Almeida et al., 2024). This integration reflects a shift towards more patient-centric healthcare models, where understanding the patient’s experience directly influences decision-making.

The EMA defines PROs as information about health or treatment that comes directly from patients, without being changed or interpreted by clinicians or others (Anonymous, 2013). In practice, this means that the patient’s own view is recorded as it is, in their own words or choices (Anonymous, 2013; Cappelleri et al., 2014). These data are collected using PROMs, such as questionnaires or symptom diaries, which must be carefully tested to ensure they are accurate, consistent, and suitable for the specific patient population (Anonymous, 2013). PROMs are especially useful for capturing how patients feel and function in daily life, including symptoms such as pain, tiredness, nausea, emotional and physical functioning, overall wellbeing, how well they follow their treatment, how satisfied they are with their care, and which treatment options they prefer (Anonymous, 2013; Cappelleri et al., 2014; European Medicines Agency, 2014).

Additionally, PROMs can be categorized in various ways, with one common distinction being between generic and disease-specific measures (Nilsson et al., 2016; van Gorp et al., 2023; Thompson and Ski, 2015; Jenkinson and Morley, 2016; Vach et al., 2018). Generic PROMs assess overall health-related quality of life and are applicable across a broad range of health conditions, such as the Paediatric Quality of Life Inventory (PedsQL), a widely-used instrument that measures physical, emotional, social, and school functioning across various age groups (Pais-Cunha et al., 2024), and PROMIS-25, a standardized PROM tool developed for children, which assesses multiple domains, including physical function, anxiety, depression, and pain interference (Niknam and Swarup, 2024). PROMs can also be grouped according to whom they are designed for, how they are meant to be used, or which aspects of health they want to measure. Disease-specific PROMs, on the other hand, are tailored to assess outcomes related to particular conditions or diseases, e.g., the Scoliosis Research Society-30 (SRS-30) instrument, specifically designed for children with spinal deformities, which evaluates pain, self-image, function, and mental health (Niknam and Swarup, 2024), or the Musculoskeletal Tumor Society (MSTS) score, used for children with musculoskeletal tumors (Baghdadi et al., 2024).

These questionnaires help to clarify the specific difficulties faced by children with specific diseases. When generic and disease-specific PROMs are used together, they provide a more complete picture of how patients are feeling and functioning. This combined approach enables comparisons across different conditions, while providing detailed information about the impacts of specific diseases (Nilsson et al., 2016; van Gorp et al., 2023). When PROMs are used according to existing guidelines, researchers and clinicians can effectively utilize them to assess patient outcomes and to inform treatment choices (Benson, 2020; Croudace et al., 2016; Velikova et al., 2017; Holmes and Briffa, 2016; Nguyen et al., 2021; Kunert et al., 2016; Kluzek et al., 2022; Al Sayah et al., 2021).

PROMs are also important in post-approval processes such as Health Technology Assessment (HTA) and Real-World Evidence (RWE) generation. HTA bodies increasingly rely on PROM data to inform pharmacoeconomic evaluations (cost-effectiveness, cost-benefit, cost-utility, cost-minimization) and to assess the value of therapies from a patient-centred perspective. By incorporating them into HTA frameworks, decision-makers can better assess the impact of treatments on patients’ quality of life and daily functioning. This data can be used to support reimbursement and policy decisions and to benchmark and improve healthcare service delivery (Jeyaraman et al., 2025; Nair and Venkatesh, 2023).

RWE further increases the utility of PROMs by capturing patient experiences outside the controlled settings of RCTs, providing insights into how treatments perform in diverse populations under routine clinical conditions (Costa et al., 2025; Mikl et al., 2024). This information is specially important for understanding long-term treatment effectiveness, safety over time, and whether patient are able to follow their treatment as prescribed. RWE from sources like electronic health records complements PROM data to fill evidence gaps (Jonker et al., 2022), particularly in rare diseases or in small patient groups that are often under-represented in trials. Using PROMs both before a medicine is approved and after it is on the market helps to ensure treatments meet patient needs, support more informed decisions, and support outcome-focused care.

### 3.2 Application of PROMs in post-marketing drug safety monitoring

PROMs are essential tools for assessing the effectiveness and safety of medications from the patient’s perspective, providing valuable insights into the patient experience that can complement clinical data and improve overall medication safety (Vrijens et al., 2024).

PROMs allow for the capture of patient-perceived AEs, assess the impact of medications on quality of life, identify potential safety issues that may not be apparent through clinical measures alone and facilitate shared decision-making between healthcare providers and patients.

In paediatric care, PROMs are particularly important for ensuring medication safety due to the unique challenges faced by this population. They can help identify age-specific side effects and safety concerns, provide insights into the impact of medications on child development and daily activities, enable better communication between children, parents, and healthcare providers about medication experiences and support personalized treatment approaches for paediatric patients (Ryan et al., 2024; Jones-Oguh et al., 2023).

In the realm of pharmacovigilance, particularly for paediatric patients, PROMs offer a systematic and thorough method of monitoring drug safety, complementing spontaneous reporting systems and helping to ensure a more comprehensive understanding of AEs in children (Lei et al., 2024). They are designed to capture the patient’s voice in clinical settings and research, providing valuable insights into the effectiveness and safety of healthcare interventions from the patient’s perspective (Cappelleri et al., 2014). This is particularly important in paediatrics, where AEs might differ from those in adults and may be less easily recognized by caregivers or clinicians. In post-marketing surveillance, PROMs are valuable for tracking drug safety and effectiveness in real-world use. By capturing patient perspectives, they help identify safety issues earlier and support quicker, more informed responses by regulators and healthcare providers.

The value of PRO data for developing improved therapies is widely acknowledged, but regulators seldom grant PRO claims. A review of RCTs revealed that PROs were consistently used as secondary or exploratory endpoints rather than primary ones, highlighting a missed opportunity to center patient experiences in therapeutic evaluations (Efficace et al., 2022). These trends suggest that while PROs are becoming more common as secondary measures, their role as primary outcomes remains rare.

To ensure that regulators grant PRO claims, sponsors should prioritize the use of validated PROMs tailored to assess concepts relevant to the target population and disease. This includes treatment-related symptoms, impact on functioning, and health-related quality of life (HRQoL). Structured approaches to collecting and analyzing PRO data, such as those outlined in regulatory guidance, can enhance methodological rigor and improve acceptance by authorities (Matts et al., 2022; Mercieca-Bebber et al., 2018).

PROMs provide a valuable means of collecting patient safety data that supports pharmacovigilance. In recent years, there has been growing recognition of the importance of PROs in monitoring drug safety. This is evident through initiatives such as the Patient-Reported Outcomes Safety Event Reporting (PROSPER) Consortium, which has developed guidance on integrating PRO data into adverse event reporting to enhance the inclusion of patients’ voices in pharmacovigilance (Banerjee et al., 2013). By addressing the limitations of traditional reporting methods, PROSPER highlights how PROMs can capture real-world safety data across diverse populations and stages of the drug lifecycle, including impacts on HRQoL that are often missed by clinician-reported outcomes (Banerjee et al., 2013). These developments underscore the feasibility and value of incorporating PROMs into routine pharmacovigilance processes, reinforcing their potential to enrich post-marketing surveillance with more patient-centred insights.

Pharmacovigilance legislation from regulatory authorities in the EU and the USA requires the inclusion of patient-reported data in the benefit-risk assessment of a pharmaceutical product (Banerjee et al., 2013; Inacio et al., 2018; Montanari-Vergallo, 2013; European Medicines Agency, 2025a). A review of European Public Assessment Reports (EPARs) from 2017 to 2022 revealed that PROs were considered in only 48.3% of authorized medicines and 52.6% of refused medicines (Meregaglia et al., 2023). Furthermore, PROs were predominantly used as secondary (53.3%) or exploratory endpoints (18.8%), with primary endpoint use remaining rare. This limited integration reflects broader challenges, including methodological concerns, the selection of appropriate PROMs, and the interpretation of results. Similarly, EMA’s analysis of oncology trials between 2017 and 2020 found that only 17.2% of indications included PRO claims in the Summary of Product Characteristics (SmPC), despite PROMs being included in 78.1% of confirmatory studies supporting submission (Teixeira et al., 2022).

The EMA has taken steps to address these gaps through initiatives like its “Regulatory Science Strategy to 2025″, which promotes systematic incorporation of PROs into risk-benefit evaluations for medicines, recognising that PRO data can complement traditional safety and efficacy measures by capturing patient-centred outcomes, such as HRQoL, treatment-related symptoms, and functional impact (European Medicines Agency, 2025b). However, barriers such as missing data, study design limitations (e.g., reliance on open-label trials), and suboptimal PROM selection continue to hinder broader adoption (European Medicines Agency, 2025b). For instance, while the U.S. FDA encourages the inclusion of PRO instruments in medical device evaluations, a study found that only 34% of authorisations used PROs as primary or secondary endpoints, reflecting their limited integration into pivotal trials (Matts et al., 2022).

A systematic review categorised PROMs based on their ability to detect intentional and unintentional non-adherence, highlighting their utility in identifying barriers such as impaired dexterity or negative treatment beliefs. These factors are critical for understanding patient behaviours influencing AEs, particularly in primary care, where intentional non-adherence is more common (Fahrni et al., 2022). PROMs also capture patient-reported experiences that clinicians may overlook, such as the personal impact of AEs, medication errors, and near misses, thus enhancing signal detection in pharmacovigilance systems (Harmark et al., 2016).

Although PROMs are already used in pharmacoepidemiological studies during the post-marketing phase, their value remains underestimated in routine pharmacovigilance practice. They are still rarely integrated as tools of interest within pharmacovigilance centres or at the point of care, where patient perspectives could meaningfully contribute to identifying and monitoring medicine-related safety concerns. Structured PROM collection systems can further amplify the patient’s voice by capturing subtle and significant AEs that may go unreported in traditional surveillance approaches.

### 3.3 Methodological foundations and measurement properties of PROMs

The evaluation and characterisation of the measurement properties of existing PROMs are guided by a consensus-based framework designed to ensure the scientific robustness of these instruments. This process is essential for selecting health measurement tools that are reliable and applicable across diverse clinical and research settings. Among the most recognised frameworks is the Consensus-based Standards for the Selection of Health Measurement Instruments (COSMIN) panel, which provides a comprehensive taxonomy of measurement properties and sets standardised criteria for their evaluation (Mokkink et al., 2019) (Table 1). PROMs are evaluated based on key dimensions: validity, reliability, responsiveness, and cross-cultural applicability. These properties ensure that PROMs are robust, reliable, and meaningful in their intended contexts. Each dimension involves specific criteria that help assess how well a PROM captures relevant health outcomes and performs consistently across various populations and settings (Davidson and Keating, 2014; Harris et al., 2016).

**TABLE 1:** Measurement properties of Patient-Reported Outcomes based on the COSMIN framework (adapted from Mokkink et al. (2019)).

Measurement properties			Definition
Validity	Content validity		• Relevance, comprehensiveness and comprehensibility of items evaluated by patients and professionals.
	Content validity	Face validity	• The extent to which a PROM looks, on first impression, to measure what it intends to.
	Construct validity		• Consistency of PROM scores with hypotheses about internal relations, relationships with other instruments, or differences among relevant groups.
	Construct validity	Structural Validity	• Assesses whether items accurately represent the underlying construct; • Reflective models require factor analysis and formative models do not require item correlation.
		Hypothesis-testing	• Evaluates the extent to which the results can be generalized to other populations or settings
		Cross-cultural validity	• Investigate whether items of a PROM behave similarly in different populations, for example, in different ethnicities or language groups, other genders or age groups or different disease populations.
	Criterion validity		• As PROMs measure constructs that can only be reported by the patients, no gold standards exist for these measures. The only exception is the extended version when investigating a short version of the same PROM.
Reliability			• Assesses the consistency and reproducibility of the measure
Reliability	Internal Consistency		• The degree of interrelatedness among items within a scale.
	Measurement error		• A PROM's systematic and random error reflects the degree to which the observed score deviates from the true score.
Responsiveness			• Ability of a PROM to detect clinically important changes over time, even if those changes are small.
Interpretability			• The degree to which one can assign qualitative meaning to quantitative scores.

The measurement properties can be assessed using various statistical and methodological approaches (Davidson and Keating, 2014; Harris et al., 2016), ensuring PROMs’ scientific rigour and applicability in clinical practice and research (Davidson and Keating, 2014; Harris et al., 2016; Hoglund et al., 2023; Koke et al., 2023). Selecting appropriate instruments is critical, requiring PROMs to be valid, reliable, and relevant to the specific health condition or treatment under investigation. Typically administered through self-completed questionnaires, PROMs are often used pre- and post-treatment to evaluate changes in PROs (De Rosis et al., 2021). Advances in digital data collection have improved the administration and analysis of PROMs, enabling real-time reporting, standardised analytics, and data visualisation. When integrated into healthcare systems, these tools facilitate meaningful interpretation and tracking of treatment impact over time (Benson, 2020; De Rosis et al., 2021).

In paediatric pharmacovigilance, PROMs support monitoring AEs by capturing dimensions such as pain interference, fatigue, and physical functioning (Carlberg Rindestig et al., 2021). Their responsiveness enables longitudinal tracking of changes in patient-reported outcomes while offering a comprehensive assessment of medication impact across physical, psychological, and social domains of a child’s quality of life (Azevedo and Lopes, 2024). One of the primary challenges in adapting adult PROMs to paediatric contexts is accounting for children’s cognitive abilities and understanding of health concepts, making it challenging to create appropriate measures for all paediatric patients (Lamparyk et al., 2021). This variability affects the reliability and validity of self-reported measures, especially in younger children (Lamparyk et al., 2021). Parent or caregiver proxy reports are often necessary for younger children or those unable to self-report. However, proxy reports may not accurately reflect the child’s internal experiences, particularly for subjective domains like pain or emotional wellbeing, leading to measurement inaccuracies in the paediatric pharmacovigilance context (Lamparyk et al., 2021). Additionally, adult PROMs often require substantial adaptation and validation to suit paediatric populations, with cultural and linguistic differences further complicating their use across diverse settings (Tan et al., 2023; Bele et al., 2023).

Data collection methods for PROMs can be broadly categorised into self-administered or healthcare professional-administered approaches. Self-administered methods, such as questionnaires, scales, and diaries, are commonly used in paper-based (Moran et al., 2023) and electronic formats (Stamp et al., 2022). Healthcare professional-administered approaches, including interviews conducted in person or online (Gunawan et al., 2022), allow for more in-depth data collection. This latter approach may be beneficial in contexts involving complex or sensitive topics, as it will enable healthcare professionals to probe for additional detail and clarification. While self-administered tools offer efficiency and scalability, professionally administered methods provide more nuanced insights, complementing both approaches in capturing comprehensive patient-reported data. The choice of method should consider factors such as the target population, the complexity of the information needed, and available resources. Furthermore, electronic data capture is increasingly used for collecting PROMs, offering advantages in accessibility, standardization, and integration with clinical systems (Wong et al., 2017; Heath et al., 2022). Some healthcare systems have implemented electronic PROM (e-PROM) platforms that link patient-reported data to clinical registries, enabling comprehensive analysis and actionable feedback to improve patient care and outcomes (Duncanson et al., 2020).

To maximise the utility of PROMs collected in routine care, it is essential to ensure that their integration into electronic systems follows standards that support both clinical use and secondary research. In this context, data standardization through the Observational Medical Outcomes Partnership (OMOP) Common Data Model (CDM) offers significant potential to enhance medication safety (Katsch et al., 2023; Dimitriadis et al., 2021). Originally developed to enable large-scale observational research, the OMOP CDM provides a robust framework for harmonising diverse healthcare data and ensuring consistent representation of clinical concepts. Incorporating PROMs into this framework not only facilitates cross-setting comparability of patient-reported experiences but also ensures that data collected in routine pharmacovigilance practice can be reused for pharmacoepidemiological studies. Furthermore, an OMOP CDM-based pharmacovigilance pipeline that includes PROMs has the potential to improve the detection of AEs signals using real-world data, contributing to a more comprehensive and efficient safety monitoring system (Shin and Lee, 2021).

## 4 Use cases: applying Ped-PRO-CTCAE in routine paediatric pharmacovigilance

The Paediatric Patient-Reported Outcomes version of the Common Terminology Criteria for Adverse Events (Ped-PRO-CTCAE) is a PROM developed by the National Cancer Institute to systematically capture symptomatic AEs directly from paediatric patients undergoing cancer treatment (Reeve et al., 2020). This tool adapts the adult PRO-CTCAE framework to the unique needs of children and adolescents, addressing documented limitations in clinician-reported AEs documentation, which often underestimates or misses subjective symptoms such as pain, fatigue, and psychological distress (Freyer et al., 2022).

Developed through collaboration with paediatric patients, parents, and clinicians, Ped-PRO-CTCAE ensures age-appropriate language and relevance to the paediatric treatment experience, permitting self-reporting by children and adolescents aged 7–17 years or, when self-reporting is not feasible, caregiver-reporting using the Ped-PRO-CTCAE Caregiver module. The instrument comprises a library of 130 items covering 62 symptomatic AE terms, grouped across multiple domains such as pain, fatigue, gastrointestinal symptoms, mood and anxiety, dermatologic symptoms, sleep disturbances, and sensory changes. Conditional branching logic can be implemented with electronic data capture, thereby reducing respondent burden. Each AE term is assessed individually through up to four standardised item formats: presence (yes/no), frequency (how often), severity (how bad), and interference (impact on daily activities). These responses are then mapped onto a grading system aligned with the CTCAE, allowing the attribution of a grade that reflects the clinical relevance of the symptom. For instance, the domain “pain” is evaluated through three items–frequency (“how often did you have pain?”), severity (“how bad was your pain?”), and interference (“how much did pain keep you from doing things you usually do?”). Responses to these items, expressed in predefined categories (e.g., Never, Sometimes, Very bad, A whole lot), are combined to derive a grade from 0 to 3 (Table 2). Grade 0 indicates the absence of the symptom (e.g., no pain); Grade 1 corresponds to mild symptoms; Grade 2 reflects moderate symptoms with limitations in instrumental activities of daily living; and Grade 3 indicates severe symptoms that interfere with self-care. The recommended recall period for each assessment is the past 7 days, ensuring temporal accuracy when capturing symptomatic adverse events. In clinical contexts, repeated weekly assessments are often advised during critical treatment periods, such as the first chemotherapy cycles, or at other key clinical milestones based on the anticipated toxicity profile of the regimen.

**TABLE 2:** Paediatric Patient-Reported Outcomes version of the Common Terminology Criteria for Adverse Events (Ped-PRO-CTCAE) attributes and item structures (adapted from the National Cancer Institute (National Cancer Institute, 2025a)).

Ped-Pro-Ctcae structures			
Frequency	Severity	Interference	Presence / Absence
How often did you have _____?	How bad was your _____?	How much did _____ keep you from doing things you usually do?	Did you have _____?
• Never • Sometimes • Most of the time • Almost all the time	• Did not have any • A little bad • Bad • Very bad	• Not at all • Some • A lot • A whole lot	• No • Yes • I don't know

Lastly, psychometric validation studies have demonstrated the instrument’s reliability, internal consistency, and construct validity in paediatric oncology populations. Its integration into pharmacovigilance systems offers a transformative opportunity to enhance AE detection, particularly for symptoms that are poorly captured through traditional spontaneous reporting mechanisms (European Medicines Agency, 2024).

Five hypothetical use scenarios were defined to illustrate different approaches for integrating the Ped-PRO-CTCAE tool into routine pharmacovigilance, outside the context of clinical trials. Each scenario demonstrates how this PROM can be operationalised to systematically collect structured symptom data reported by children or caregivers, with the ultimate goal of improving the quality of drug safety monitoring in real-world settings.

### 4.1 Adaptive integration into the spontaneous reporting form

Incorporating Ped-PRO-CTCAE into existing spontaneous reporting platforms could significantly enrich pharmacovigilance data quality. When a reporter submits a suspected AE report involving symptoms covered by Ped-PRO-CTCAE (e.g., pain, anxiety, sleep disturbances) associated with paediatric oncology treatment, the system could automatically prompt the completion of relevant PROM items (Lambert et al., 2024). This conditional logic would operate within the reporting form, ensuring seamless integration without overwhelming users (National Cancer Institute, 2025b; Montgomery et al., 2022). Evidence shows that e-PROM platforms improve real-time symptom detection and data accuracy in paediatric populations (National Cancer Institute, 2025b). This integration represents a practical step towards evolving spontaneous reporting systems from purely passive tools into more dynamic pharmacovigilance mechanisms that support longitudinal follow-up. Moreover, it promotes harmonised monitoring by enabling standardised symptom assessment across patients through a validated instrument.

Use scenario 1: A 10-year-old boy undergoing high-dose methotrexate chemotherapy for osteosarcoma presents with persistent vomiting. A paediatric oncologist submits a spontaneous report through the national pharmacovigilance platform, indicating the AE and selecting methotrexate as the suspected medication. Because “nausea” and “vomiting” are Ped-PRO-CTCAE domains and the presence of a suspected oncological treatment is identified (either through the reported therapeutic indication or comorbidity), and provided that the reported symptom onset falls within the 7-day recall period required by the instrument, the system automatically prompts the completion of the relevant PROM items. The caregiver, together with the child, completes the questionnaire electronically, providing structured data on severity, frequency, and functional impact, including interference with eating and school attendance. These data, collected via predefined Ped-PRO-CTCAE items, allow refined grading of the AE (e.g., Grade 3 vomiting due to significant daily life disruption). Once submitted, the pharmacovigilance team performs case triage, where MedDRA coding is applied based on the reported event terminology and relevant PROM-derived details. The PROM is re-administered 2 weeks later to monitor recurrence before the next chemotherapy cycle, adding valuable temporal resolution and supporting longitudinal case management.

### 4.2 Post-notification deployment in selected cases

An alternative or complementary strategy to integrating Ped-PRO-CTCAE directly into spontaneous reporting forms is the use of PROMs by pharmacovigilance centres during follow-up processes. Upon receiving a paediatric AE report associated with oncological treatment, pharmacovigilance professionals can assess whether the case involves symptoms that fall within the scope of Ped-PRO-CTCAE domains and whether the reported symptom onset falls within the instrument’s required 7-day recall period (Freyer et al., 2022). If applicable, trained staff may initiate a targeted follow-up using the PROM to capture the patient’s or caregiver’s direct account of symptom burden and severity.

Administration methods can vary depending on local infrastructure and age-appropriateness. Self-report is feasible for children aged 7–17 years, while caregiver proxies may be used for younger patients (Reeve et al., 2020). The tool can be applied via telephone interviews, digital survey links, or, where possible, during in-person follow-up appointments (Montgomery et al., 2022). This flexibility facilitates use in multiple care settings, although implementation may face practical constraints related to staff time, training, and data integration (Minasian et al., 2022).

The added value of this approach lies in the documented discrepancies between clinician-reported CTCAE grades and child self-reports. For example, one study found that clinicians underreported 63% of symptomatic AEs compared to paediatric self-reports, while caregivers tended to overreport by 41% (Freyer et al., 2022). Ped-PRO-CTCAE thus helps correct this imbalance by capturing subjective symptoms that might otherwise be missed.

Optimal implementation aligns the PROM with treatment timelines, ideally initiating with a baseline assessment followed by weekly or biweekly evaluations during high-toxicity periods such as chemotherapy induction (Reeve et al., 2020; National Cancer Institute, 2025b). Integration into EHRs or use via standalone applications can automate reminders and streamline data flow for pharmacovigilance teams (Lambert et al., 2024; National Cancer Institute, 2025b).

Unlike the embedded solution described in scenario 1, this strategy requires a manual post-reporting action by a pharmacovigilance professional. Although more resource-intensive, it offers greater selectivity, allowing targeted deployment of Ped-PRO-CTCAE in cases where added symptom granularity is clinically relevant. However, the delay between initial report and PROM administration may increase recall bias and reduce immediacy of data capture.

Use scenario 2: A 13-year-old girl with acute myeloid leukaemia undergoing consolidation therapy with high-dose cytarabine is reported through the spontaneous reporting system as experiencing “severe malaise”. The case reaches the regional pharmacovigilance centre, where the vague symptom prompts further clarification. Upon contacting the caregiver, it becomes evident that the child has been experiencing persistent fatigue starting 3 days prior to the contact. As “fatigue” is a domain included in Ped-PRO-CTCAE, the centre initiates a telephone-based administration of the relevant items. The child reports moderate fatigue affecting school concentration and daily functioning, which corresponds to a Grade 2 AE. These specific dimensions, such as interference with daily activities and recall-based frequency of symptoms over the past week, are captured through validated Ped-PRO-CTCAE language. The PROM is scheduled again 2 weeks later to evaluate symptom evolution under continued treatment. This use supports causality assessment and enhances the clinical relevance of the pharmacovigilance report, particularly in cases with ambiguous initial terminology.

### 4.3 Routine clinical integration during paediatric treatment pathways

The Ped-PRO-CTCAE is optimally administered during routine clinical follow-up visits for children receiving high-risk therapies, such as chemotherapy or immunomodulatory treatments. Healthcare teams can embed the PROM within standardised clinical workflows, ensuring systematic data collection at predefined intervals (e.g., pre-treatment, weekly during active therapy, and post-treatment). Integration with EHR systems is critical for scalability and usability, as demonstrated by studies showing that EHR-embedded PROMs improve clinician access to symptom data and facilitate real-time decision-making. For example, Northwestern Medicine’s EHR-integrated PRO system enabled automated triage for psychosocial care and enhanced patient-provider communication by displaying symptom trends directly in clinical dashboards (Zhang et al., 2019). Similarly, the PROTEUS-Practice Guide emphasises that full EHR integration allows PROM data to populate discrete fields, enabling longitudinal tracking and reducing documentation redundancy (Proteus Consortium, 2025).

However, current EHR systems often lack bidirectional connectivity with pharmacovigilance databases. Data collected via Ped-PRO-CTCAE in clinical settings remains siloed within the EHRs unless manually extracted and reported to regulatory agencies. This limitation mirrors broader challenges in pharmacovigilance, where only 5%–10% of AEs documented in EHRs are voluntarily reported through spontaneous reporting systems (Alloush et al., 2024). EHRs prioritise clinical care documentation over regulatory compliance, and pharmacovigilance systems like the FDA’s FAERS or the EU’s EudraVigilance require structured and validated reports that clinicians often lack time to prepare (Muzaffar et al., 2023).

Some countries have pioneered partial integration between EHRs and pharmacovigilance systems to mitigate these gaps. In the Netherlands, the Jeroen Bosch Hospital implemented a systematic AE registration module within its EHRs, which automatically flags severe or unexpected reactions for review by pharmacovigilance teams. During a follow-up period of a year, this system identified 1243 AEs, including 217 previously unrecorded drug-event associations (Alloush et al., 2024). Similarly, the UK’s NHS has tested Fast Healthcare Interoperability Resources-based interfaces to map EHR-derived symptom data to standardised MedDRA terms, enabling semi-automated AE reporting. These initiatives leverage NLP to extract PROM responses and clinician notes, though human validation remains necessary to ensure accuracy (Kim et al., 2024).

To maximize Ped-PRO-CTCAE’s pharmacovigilance utility, healthcare institutions should develop, for example, EHR-PROM modules with built-in regulatory reporting flags, such as auto-populated fields for seriousness criteria (e.g., hospitalization, disability) and suspected drug associations, aligning, for example, with the FDA’s Sentinel Initiative, which uses distributed EHR networks for active drug safety surveillance or even leverage existing pharmacovigilance frameworks, where EHR-derived AEs are reviewed alongside spontaneous reports to identify emerging safety signals (Alloush et al., 2024; Muzaffar et al., 2023).

Use scenario 3: A 14-year-old boy receiving maintenance therapy for acute lymphoblastic leukaemia attends his scheduled hospital visit for methotrexate and vincristine administration in a day-care oncology unit. Shortly after the start of the infusion, as part of routine clinical monitoring, he is invited to complete the Ped-PRO-CTCAE questionnaire on a tablet. On this occasion, he reports severe abdominal pain and moderate fatigue, both interfering with appetite and school performance–two domains specifically captured by the Ped-PRO-CTCAE. Based on the reported severity and functional impact, these correspond to a Grade 3 AE for abdominal pain and a Grade 2 AE for fatigue. His responses are automatically saved into the EHR, where they are reviewed by the gastroenterologist during the same visit. The clinical team considers this information when evaluating treatment tolerability and planning future dosing. In hospitals with a pharmacovigilance liaison, the PROM data are also used to trigger a draft AE report. Where no such liaison exists, the treating team may initiate the report themselves. This systematic and scheduled use of Ped-PRO-CTCAE during high-risk treatments enables timely detection of symptomatic toxicities and supports both clinical and regulatory safety monitoring.

### 4.4 Integration into ambulatory pharmacy workflows

Hospital outpatient pharmacies can represent a valuable opportunity to collect patient-reported outcomes, particularly in children receiving high-risk therapies such as oral chemotherapies, biologics, or immunosuppressants. When patients attend the pharmacy to initiate a new treatment cycle or renew an ongoing prescription, this visit can serve as an opportune point for systematically capturing symptom data experienced during the previous 7 days, which corresponds to the validated recall period of the Ped-PRO-CTCAE. Pharmacy staff may invite the caregiver and, when appropriate, the child to complete the relevant items using a tablet or secure digital platform, allowing the collection of structured and standardised data without requiring an additional clinical appointment. Nevertheless, the value of integrating PROM collection at the dispensing point depends strongly on timing, as its usefulness is maximised when pharmacy visits are synchronised with treatment renewal cycles or periods of higher risk for treatment-related adverse events, ensuring that the collected information remains clinically relevant and aligned with the instrument’s intended scope (Crossnohere et al., 2024).

Evidence from paediatric oncology shows that PROMs administered in ambulatory settings reveal symptoms like pain, fatigue, or functional impairments that are frequently underdetected by clinicians (Meryk et al., 2022). However, as observed in Dutch and UK healthcare systems, data captured through PROMs in pharmacy or clinical settings rarely flow automatically into pharmacovigilance systems (Bele and Santana, 2025). This creates a missed opportunity to enhance post-marketing surveillance with structured real-world symptom data.

Use scenario 4: A 9-year-old girl with acute lymphoblastic leukaemia is undergoing maintenance treatment with oral 6-mercaptopurine at home. Every 28 days, she and her caregiver visit the hospital’s outpatient pharmacy to collect the next cycle of medication. During dispensing, the pharmacy team invites them to complete a Ped-PRO-CTCAE module on a tablet. Their responses indicate moderate fatigue and episodes of low-grade fever over the past 3 days–symptoms that had not been previously reported. These domains, specifically “fatigue” and “fever,” are captured with structured metrics such as severity and interference with daily function, enabling early identification of potential toxicities like febrile neutropenia, known to be associated with 6-mercaptopurine. The pharmacist enters the results into the patient’s EHR and submits an individual case safety report to the national pharmacovigilance system. This application demonstrates how integrating Ped-PRO-CTCAE into dispensing workflows provides timely safety insights without requiring extra clinic visits.

### 4.5 Structured symptom monitoring during medication rechallenge

The strategic use of Ped-PRO-CTCAE during medication rechallenge, defined as the intentional re-administration of a drug after an AE, provides a structured framework for monitoring symptom recurrence and supporting causality assessment in paediatric populations (Stanulovic et al., 2013).

Rechallenge decisions in children carry inherent risks, as they may develop new or more severe AEs, and by embedding Ped-PRO-CTCAE into rechallenge protocols, clinicians and pharmacovigilance teams can collect baseline patient-reported data before re-exposure and compare it with follow-up assessments, objectively tracking changes in severity, frequency, and functional impact of symptoms. This enables the identification of worsening grades or the emergence of new events after drug reintroduction, which can help differentiate potential drug-related effects from background symptoms and mitigate risks associated with re-exposure. This approach can be initiated by the clinical team during consultations, via EHR alerts, or by pharmacovigilance units for previously reported cases, ensuring comprehensive monitoring and supporting evidence-based decision-making for high-risk paediatric patients (Reeve et al., 2020).

Use scenario 5: A 6-year-old boy with Hodgkin lymphoma had previously developed a diffuse rash and low-grade fever following his third doxorubicin infusion. After resolution and clinical reassessment, the decision is made to cautiously reintroduce the drug. On the day of rechallenge, the caregiver and child are invited to complete the relevant Ped-PRO-CTCAE items, focusing on domains such as “rash,” “fever,” and “general discomfort.” The PROM is re-administered 24 h and 7 days after the infusion. While the rash does not recur, the child reports moderate fatigue interfering with daily activities (Grade 2 AE) and persistent low-grade fever (Grade 1 AE), both newly reported compared to the baseline assessment. These structured before-and-after data allow the clinical team to evaluate whether changes in symptom profiles are temporally associated with drug re-exposure and to prioritise appropriate follow-up. Importantly, although the fever was ultimately attributed to an intercurrent viral infection unrelated to the chemotherapy, the Ped-PRO-CTCAE provides standardised, patient-reported metrics on symptom evolution that enrich clinical decision-making. The pharmacovigilance team updates the initial spontaneous report with these structured data, contributing to a more informative rechallenge report and facilitating a better-supported causality assessment.

### 4.6 Strengths and limitations of the proposed use scenarios

The five proposed scenarios highlight both the potential and the challenges of integrating PROMs into paediatric pharmacovigilance. Among the key strengths, PROMs enable structured and standardised symptom collection, provide validated grading of adverse events, and capture patient-centred data that are often underreported in traditional spontaneous reporting systems. By harmonising the assessment of subjective symptoms, these tools improve data granularity and support more consistent safety evaluations across clinical and real-world settings. In addition, PROMs introduce temporal resolution into safety monitoring, enabling comparisons between baseline and follow-up assessments, which is particularly valuable in contexts such as rechallenge or longitudinal follow-up. Finally, PROM-derived data are inherently complementary to clinician-reported outcomes, helping to reveal discrepancies and capture symptomatic toxicities that might otherwise be overlooked.

However, several limitations must be acknowledged. First, PROMs do not establish causality; they generate structured patient-reported data that require clinical and pharmacovigilance interpretation, particularly when background symptoms coexist with treatment-related effects. Second, their successful implementation depends on the completeness and quality of initial adverse event reports, many of which lack essential contextual details such as therapeutic indication, comorbidities, or suspected drug–disease relationships. In these cases, automated PROM triggers may fail to activate, especially in multi-indication or off-label scenarios. Third, despite their methodological value, PROMs introduce operational challenges related to staff training, resource allocation, and interoperability across systems. Interoperability with existing electronic health records is often hampered by heterogeneous data models and the lack of standardised pipelines to pharmacovigilance databases, so PROM data remain siloed unless actively extracted and transformed. In addition, data privacy and consent requirements in paediatric populations, together with variable caregiver health literacy, may limit both the willingness and the ability of families to provide accurate symptom reports. Potential mitigation strategies include phased implementation in high-risk settings, targeted training for clinicians and pharmacovigilance staff, adoption of common data models and coding standards to support interoperability, the use of simplified and culturally adapted PROM interfaces for caregivers, and clear, tiered consent procedures that distinguish routine clinical monitoring from secondary use for research or regulatory purposes. Finally, timing is critical: the Ped-PRO-CTCAE recall period restricts its applicability to symptoms experienced within the last days, reducing its utility for late follow-up.

## 5 Concluding remarks

The integration of PROMs into paediatric pharmacovigilance represents a pivotal step towards more sensitive, timely, and patient-centred safety monitoring. By capturing subjective symptoms directly from children - or, when necessary, via caregiver-assisted reporting - PROMs complement clinician-reported data and help address the underreporting of AEs, particularly those affecting daily functioning or quality of life. When adapted to paediatric needs and embedded into clinical, regulatory, and dispensing workflows, PROMs can strengthen both individual case management and broader signal detection. Their value lies not only in enhancing the granularity of symptom documentation but also in fostering a participatory approach to medicine safety, where children’s lived experiences inform clinical and regulatory decision-making. As pharmacovigilance evolves in the era of real-world evidence, the structured and context-aware use of PROMs should be viewed not as a complementary tool, but as an essential element of safer, more responsive paediatric therapeutic strategies.
